# Stem cell library screen identified ruxolitinib as regulator of osteoblastic differentiation of human skeletal stem cells

**DOI:** 10.1186/s13287-018-1068-x

**Published:** 2018-11-21

**Authors:** Nihal AlMuraikhi, Dalia Ali, Aliah Alshanwani, Radhakrishnan Vishnubalaji, Muthurangan Manikandan, Muhammad Atteya, Abdulaziz Siyal, Musaad Alfayez, Abdullah Aldahmash, Moustapha Kassem, Nehad M. Alajez

**Affiliations:** 1 Stem Cell Unit, Department of Anatomy, College of Medicine, King Saud University, Riyadh, 11461 Kingdom of Saudi Arabia; 2 Molecular Endocrinology Unit (KMEB), Department of Endocrinology, University Hospital of Odense and University of Southern Denmark, Odense, Denmark; 3 Department of Physiology, College of Medicine, King Saud University, Riyadh, 11461 Kingdom of Saudi Arabia; 4 Histology Department, Faculty of Medicine, Cairo University, Cairo, Egypt; 5 Prince Naif Health Research Center, King Saud University, Riyadh, 11461 Kingdom of Saudi Arabia; 6 Department of Cellular and Molecular Medicine, Danish Stem Cell Center (DanStem), University of Copenhagen, 2200 Copenhagen, Denmark; 7 Cancer Research Center, Qatar Biomedical Research Institute, Hamad Bin Khalifa University (HBKU), Qatar Foundation, Doha, Qatar

## Abstract

**Background:**

Better understanding of the signaling pathways that regulate human bone marrow stromal stem cell (hBMSC) differentiation into bone-forming osteoblasts is crucial for their clinical use in regenerative medicine. Chemical biology approaches using small molecules targeting specific signaling pathways are increasingly employed to manipulate stem cell differentiation fate.

**Methods:**

We employed alkaline phosphatase activity and staining assays to assess osteoblast differentiation and Alizarin R staining to assess mineralized matrix formation of cultured hBMSCs. Changes in gene expression were assessed using an Agilent microarray platform, and data normalization and bioinformatics were performed using GeneSpring software. For in vivo ectopic bone formation experiments, hMSCs were mixed with hydroxyapatite–tricalcium phosphate granules and implanted subcutaneously into the dorsal surface of 8-week-old female nude mice. Hematoxylin and eosin staining and Sirius Red staining were used to detect bone formation in vivo.

**Results:**

We identified several compounds which inhibited osteoblastic differentiation of hMSCs. In particular, we identified ruxolitinib (INCB018424) (3 μM), an inhibitor of JAK-STAT signaling that inhibited osteoblastic differentiation and matrix mineralization of hMSCs in vitro and reduced ectopic bone formation in vivo. Global gene expression profiling of ruxolitinib-treated cells identified 847 upregulated and 822 downregulated mRNA transcripts, compared to vehicle-treated control cells. Bioinformatic analysis revealed differential regulation of multiple genetic pathways, including TGFβ and insulin signaling, endochondral ossification, and focal adhesion.

**Conclusions:**

We identified ruxolitinib as an important regulator of osteoblast differentiation of hMSCs. It is plausible that inhibition of osteoblast differentiation by ruxolitinib may represent a novel therapeutic strategy for the treatment of pathological conditions caused by accelerated osteoblast differentiation and mineralization.

**Electronic supplementary material:**

The online version of this article (10.1186/s13287-018-1068-x) contains supplementary material, which is available to authorized users.

## Background

Bone marrow stromal (also known as mesenchymal or skeletal) stem cells (BMSCs) exist within the bone marrow stromal and are capable for differentiation into mesoderm-type cells including bone-forming osteoblasts [1]. A number of signaling pathways have been implicated in regulating differentiation of human BMSCs (hBMSCs) into osteoblasts that include TGF-B [2], Wnt [3], and several intracellular kinases [4]. However, several other signaling pathways have been reported to regulate different aspects of stem cell biology in a number of stem cell systems [5] but their role in regulating hBMSC differentiation into osteoblastic cells are not well studied.

Chemical biology approaches using small molecules targeting specific intracellular or signaling factors are very important tools for studying stem cell differentiation and in vitro manipulation of stem cells (add ref). In addition, small molecules that induce stem cell differentiation are being employed as an alternative approach to classical stem cell differentiation protocols that require complex mixture of growth factors and cytokines, because of their scalable production, stability, ease of use, and low cost [6–8].

We have previously employed small molecule libraries to dissection mechanisms underlying differentiation potential of hBMSCs into osteoblasts [9] [4] and adipocytes [8].

Herein, we conducted an unbiased small molecule stem cell signaling library screen that covers several signaling pathways and identified ruxolitinib as an important regulator of osteoblast differentiation of hBMSCs.

## Materials and methods

### Stem cell signaling compound library

A stem cell signaling compound library, purchased from Selleckchem Inc. (Houston, TX, http://www.selleckchem.com) and consisted of 73 biologically active small molecular inhibitors, was employed in the presented study. An initial screen was conducted at a concentration of 3 μM.

### Cell culture

We employed a telomerized hMSC line (hMSC-TERT) as a model for hBMSCs. The hMSC-TERT line was generated through an overexpression of the human telomerase reverse transcriptase gene (hTERT). hMSC-TERT exhibits the typical features of primary hMSCs including indefinite self-renewal and multipotency, in addition to the expression of all known markers of primary hMSCs [10–12]. The cells were maintained in DMEM, a basal medium supplemented with 4500 mg/L d-glucose, 4 mM l-glutamine, and 110 mg/L 10% sodium pyruvate, in addition to 10% fetal bovine serum (FBS), 1% penicillin–streptomycin, and 1% nonessential amino acids. All reagents were purchased from Thermo Fisher Scientific, Waltham, MA (http://www.thermofisher.com). Cells were incubated in 5% CO_2_ incubators at 37 °C and 95% humidity.

### Osteoblast differentiation

The cells were cultured to 80–90% confluence and were incubated in osteoblast induction medium (DMEM containing 10% FBS, 1% penicillin–streptomycin, 50 μg/ml l-ascorbic acid (Wako Chemicals GmbH, Neuss, Germany, http://www.wako-chemicals. de/), 10 mM b-glycerophosphate (Sigma-Aldrich), 10 nM calcitriol (1a,25-dihydroxyvitamin D3; Sigma-Aldrich), and 10 nM dexamethasone (Sigma-Aldrich)). Each small molecule inhibitor was added at a concentration of 3 μM, in the osteoblast induction medium. The cells were exposed to the inhibitors throughout the differentiation period. Control cells were treated with osteoblast induction medium containing dimethyl sulfoxide (DMSO) as vehicle.

### Cell viability assay

Cell viability assay was performed using alamarBlue assay according to the manufacturer’s recommendations (Thermo Fisher Scientific). In brief, cells were cultured in 96-well plates in 200 μl of the medium for 10 days, then 20 μl of alamarBlue substrate was added, and plates were incubated in the dark at 37 °C for 1 h. Readings were taken using fluorescent mode (Ex 530 nm/Em 590 nm) using a BioTek Synergy II microplate reader (BioTek Inc., Winooski, VT, USA).

### Alkaline phosphatase activity quantification

To quantify alkaline phosphatase (ALP) activity, we employed the BioVision ALP activity colorimetric assay kit (BioVision, Inc., Milpitas, CA, http:// www.biovision.com/) with some modifications. The cells were cultured in 96-well plates. On day 10, the cells were rinsed once with phosphate-buffered saline (PBS) and fixed using 3.7% formaldehyde in 90% ethanol for 30 s at room temperature. Fixative was removed, and 50 μl of p-nitrophenyl phosphate solution was added to each well and incubated for 30–60 min. Optical densities were then measured at 405 nm using a SpectraMax/M5 fluorescence spectrophotometer plate reader. ALP enzymatic activity was normalized to cell number.

### In vivo ectopic bone formation assay

All animal experimental procedures were approved by the Animal Care Committees of King Saud University. Cells were harvested via trypsinization, washed in PBS, and resuspended in culture medium with or without ruxolitinib. Approximately 5 × 10^5^ cells were mixed with 40 mg of hydroxyapatite–tricalcium phosphate granules per each implant (HA/TCP, Zimmer Scandinavia, Albertslund, Denmark) and implanted subcutaneously into the dorsal surface of 8-week-old female nude mice, as previously described [13]. After 28 days, the implants were recovered, fixed in 4% paraformaldehyde, decalcified using formic acid solution (0.4 M formic acid and 0.5 M sodium formate), and embedded in paraffin. Tissue blocks were sectioned at 4 μm. Sections of paraffin-embedded implants were stained with hematoxylin and eosin and Sirius Red to identify areas of the formed bone.

### Alkaline phosphatase staining

Cells were stained on day 10 of osteoblast differentiation. Cells cultured in 12-well plates were washed in PBS and fixed in 10 mM acetone/citrate buffer at pH 4.2 for 5 min at room temperature. The fixative was removed, and the Naphthol/Fast Red stain [0.2 mg/mL Naphthol AS-TR phosphate substrate (Sigma)] [0.417 mg/mL of Fast Red (Sigma)] was added for 1 h at room temperature. The cells were then rinsed with water and imaged under the microscope.

### Alizarin Red S staining for mineralized matrix formation

Cells cultured in 12-well plates were stained on day 21 of osteoblast differentiation. The cells were washed twice with PBS and then fixed with 4% paraformaldehyde for 15 min at room temperature. Fixative was then removed, and the cells were washed with distilled water and stained with 2% Alizarin Red S Staining Kit (ScienceCell, Research Laboratories, Cat. No. 0223) for 20–30 min at room temperature. Subsequently, the dye was washed off with water and cells were imaged under the microscope.

### RNA extraction and cDNA synthesis

Total RNA was isolated from cell pellets after 10 and 21 days of osteoblast differentiation using the total RNA Purification Kit (Norgen Biotek Corp., Thorold, ON, Canada, https://norgenbiotek.com/) according to the manufacturer’s protocol. The concentrations of total RNA were measured using NanoDrop 2000 (Thermo Fisher Scientific). cDNA was synthesized using 500 ng of total RNA and the Thermo Fisher Scientific High Capacity cDNA Transcription Kit according to the manufacturer’s protocol.

### qRT-PCR

Expression levels of the mRNAs were validated using SYBR Green-based quantitative reverse transcriptase-polymerase chain reaction (qRT-PCR) with an Applied Biosystems ViiA™ 7 Real-Time PCR System (Thermo Fisher Scientific). Primers used in current study are listed in Table 1. The 2∆CT value method was used to calculate relative expression, and analysis was performed as previously described [14].

**Table 1:** Real-time PCR primer sequences

Gene name	Forward primer	Reverse primer
ACTB	5′AGCCATGTACGTTGCTA	5′AGTCCGCCTAGAAGCA
ALP	5′GGA ACT CCT GAC CCT TGA CC3′	5′TCC TGT TCA GCT CGT ACT GC3′
RUNX2	5′GTA GAT GGA CCT CGG GAA CC3′	5′GAG GCG GTC AGA GAA CAA AC3′
COMP	5′CCGACACCGCCTGCGTTCTT3′	5′AGCGCCGCGTTGGTTTCCTG3′
THBS2	5′TTGGCAAACCAGGAGCTCAG3′	5′GGTCTTGCGGTTGATGTTGC3′
TNF	5′ACT TTG GAG TGA TCG GCC3′	5′GCT TGA GGG TTT GCT ACA AC3′
LIF	5′GCCACCCATGTCACAACAAC	5′CCCCCTGGGCTGTGTAATAG
SOCS3	5′TTCGGGACCAGCCCCC3′	5′AAACTTGCTGTGGGTGACCA3′

### Gene expression profiling by microarray

One hundred fifty nanograms of total RNA was labeled using low input Quick Amp Labeling Kit (Agilent Technologies, Santa Clara, CA, http://www.agilent.com) and then hybridized to the Agilent Human SurePrint G3 Human GE 8 × 60 k microarray chip. All microarray experiments were performed at the Microarray Core Facility (Stem Cell Unit, Department of Anatomy, King Saud University College of Medicine, Riyadh, Saudi Arabia). The extracted data were normalized and analyzed using GeneSpring 13.0 software (Agilent Technologies). Pathway analyses were performed using the single experiment pathway analysis feature in GeneSpring 13.0 as described before [15]. Twofold cutoff and *P* (corr) < 0.05 (Benjamini–Hochberg multiple testing corrected) were used to determine significantly changed transcripts.

### Statistical analysis

Statistical analysis and graphing were performed using Microsoft Excel 2010 and GraphPad Prism 6 software (GraphPad software, San Diego, CA, USA), respectively. Results were presented as mean ± SEM from at least two independent experiments. Unpaired *t* test was used to determine statistical significance and *P* values < 0.05 was considered statistically significant.

## Results

### Stem cell signaling library screen identified inhibitors of osteoblast differentiation of hBMSCs

**Fig. 1:**
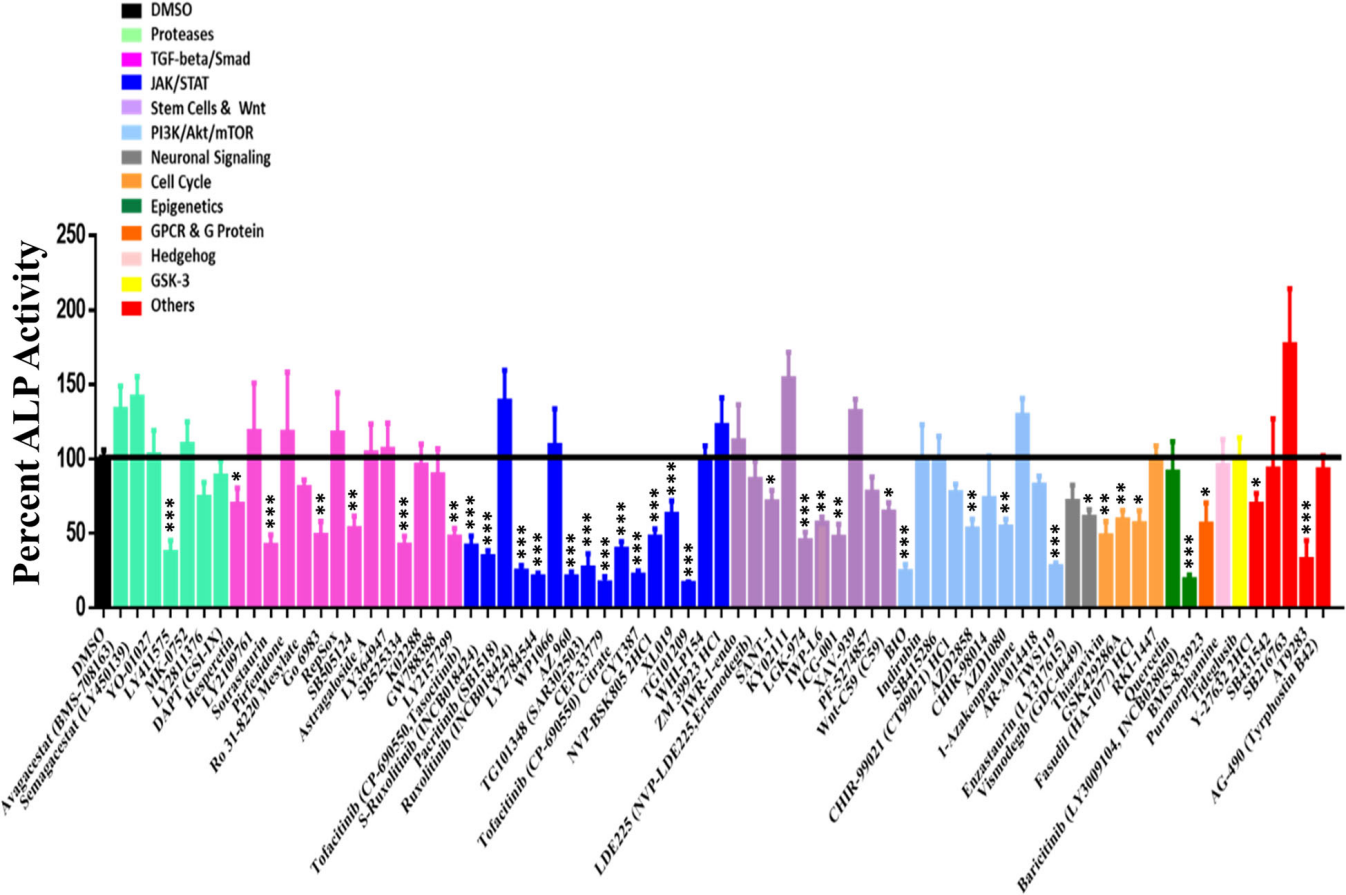
Functional screen of stem cell signaling small molecule library for their effects on osteoblast differentiation of human bone marrow stromal stem cells (hBMSCs). hBMSCs were induced into osteoblasts for 10 days in the presence of the indicated small molecule inhibitors (3.0 μM) or DMSO vehicle control. Data are presented as mean alkaline phosphatase (ALP) activity ± SEM, *n* ≥ 10 from three independent experiments. Small molecules are grouped according to their targeted signaling pathway. DMSO dimethyl sulfoxide. *P < 0.05; **P < 0.05; ***P < 0.0005

**Fig. 2:**
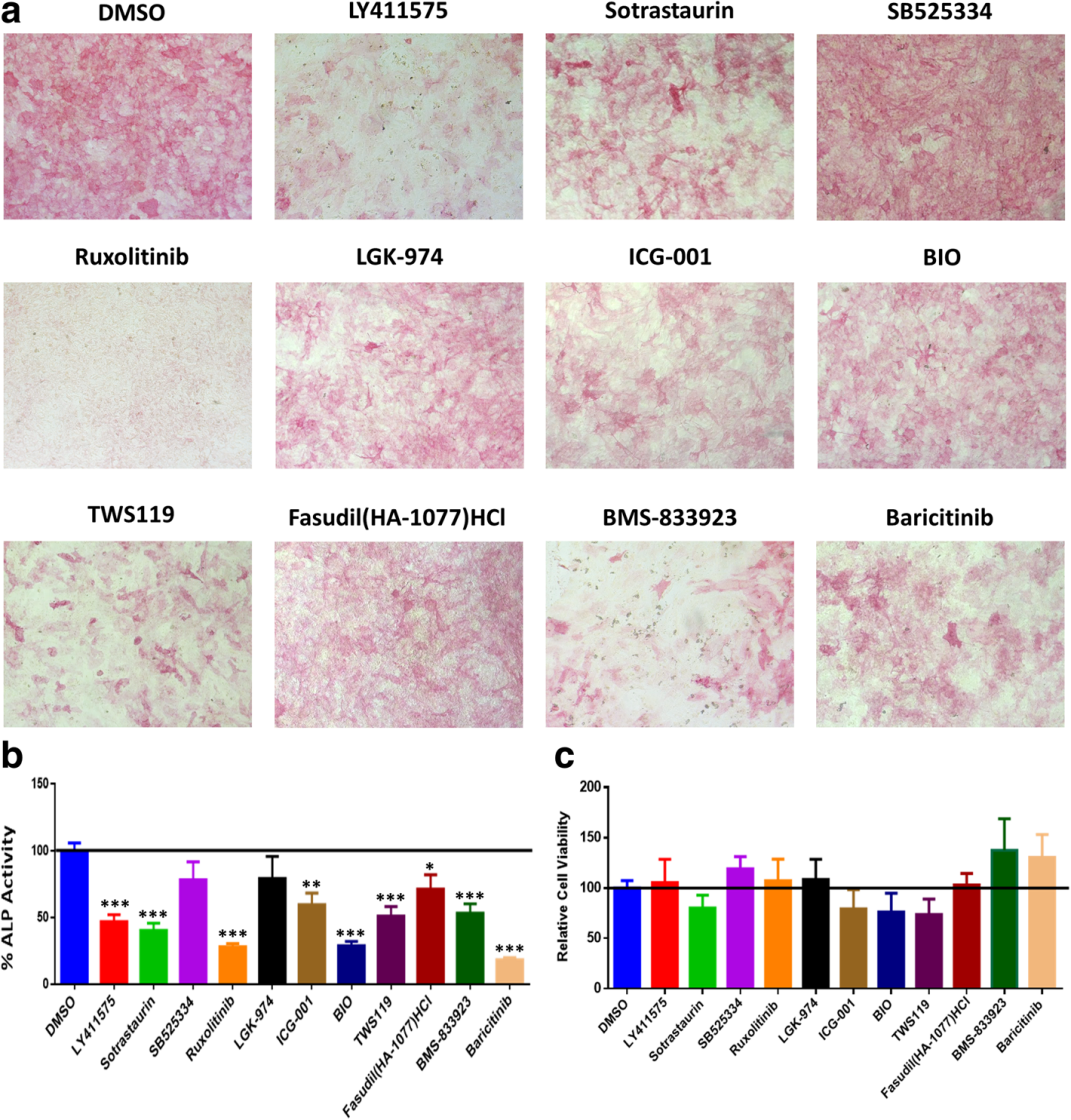
The effect of a selected panel of small molecules targeting multiple signaling pathways on osteoblast differentiation of hBMSCs. **a** Representative alkaline phosphatase (ALP) staining of hBMSCs on day 10 following treatment with the indicated compounds (concentration 3.0 μM). Images were taken at × 10 magnification using a Zeiss inverted microscope. **b** Quantification of ALP activity in hBMSCs following treatment with the indicated compounds (concentration 3 μM) versus vehicle-treated control cells at day 10. Data are presented as mean percentage ALP activity ± SEM, *n* > 16. **P < 0.05; ***P < 0.0005. **c** Cell viability assay using alamarBlue showing the relative cell viability in hBMSCs following treatment with the indicated compounds (3 μM) versus vehicle-treated control cells on day 10 post-osteoblast differentiation. Abbreviations: ALP alkaline phosphatase, DMSO dimethyl sulfoxide

A stem cell signaling library consisting of 73 chemical compounds was used for the initial screen for their effects on osteoblastic differentiation of hBMSCs using ALP activity quantification as a read-out. All small molecules were tested at a concentration 3 μM. As shown in Fig. 1, the majority of small molecules reduced ALP activity of hBMSCs. Based on this initial screen, we chose 11 compounds (ruxolitinib (INCB018424), LY411575, BMS-833923, sotrastaurin, SB525334, LGK-974, ICG-001, BIO, TWS119, fasudil (HA-1077) HCl, and baricitinib (LY3009104, INCB028050)) for follow-up studies. The name of small molecule and their molecular targets are listed in Table 2. As shown in Fig. 2, several of the tested molecules inhibited osteoblastic differentiation of hBMSCs as evidenced by reduced ALP cytochemical staining at day 10 post-osteoblast differentiation induction (Fig. 2a) and this was concordant with the reduced ALP activity (Fig. 2b). These molecules did not exert significant effects on hBMSC viability (Fig. 2c). Among these small molecules, we chose ruxolitinib (INCB018424) for more detailed studies as it yielded the most consistent and potent effect on osteoblast differentiation and its effect on osteoblast differentiation of hBMSCs has not been studied before.

**Table 2:** Characteristics of the selected 11 compounds of stem cell signaling library

Name of compound	Target	Pathway
LY411575	Gamma-secretase	Proteases
Sotrastaurin	PKC	TGF-beta/Smad
SB525334	TGF-beta/Smad	TGF-beta/Smad
Ruxolitinib (INCB018424)	JAK1/JAK2	JAK/STAT
LGK-974	Wnt/beta-catenin	Stem cells and Wnt
ICG-001	Wnt/beta-catenin	Stem cells and Wnt
BIO	GSK-3	PI3K/Akt/mTOR
TWS119	GSK-3	PI3K/Akt/mTOR
Fasudil (HA-1077) HCl	ROCK	Cell cycle
Baricitinib (LY3009104, INCB028050)	JAK	Epigenetics
BMS-833923	Hedgehog/smoothened	GPCR and G protein

### Ruxolitinib inhibits mineralized matrix formation

**Fig. 3:**
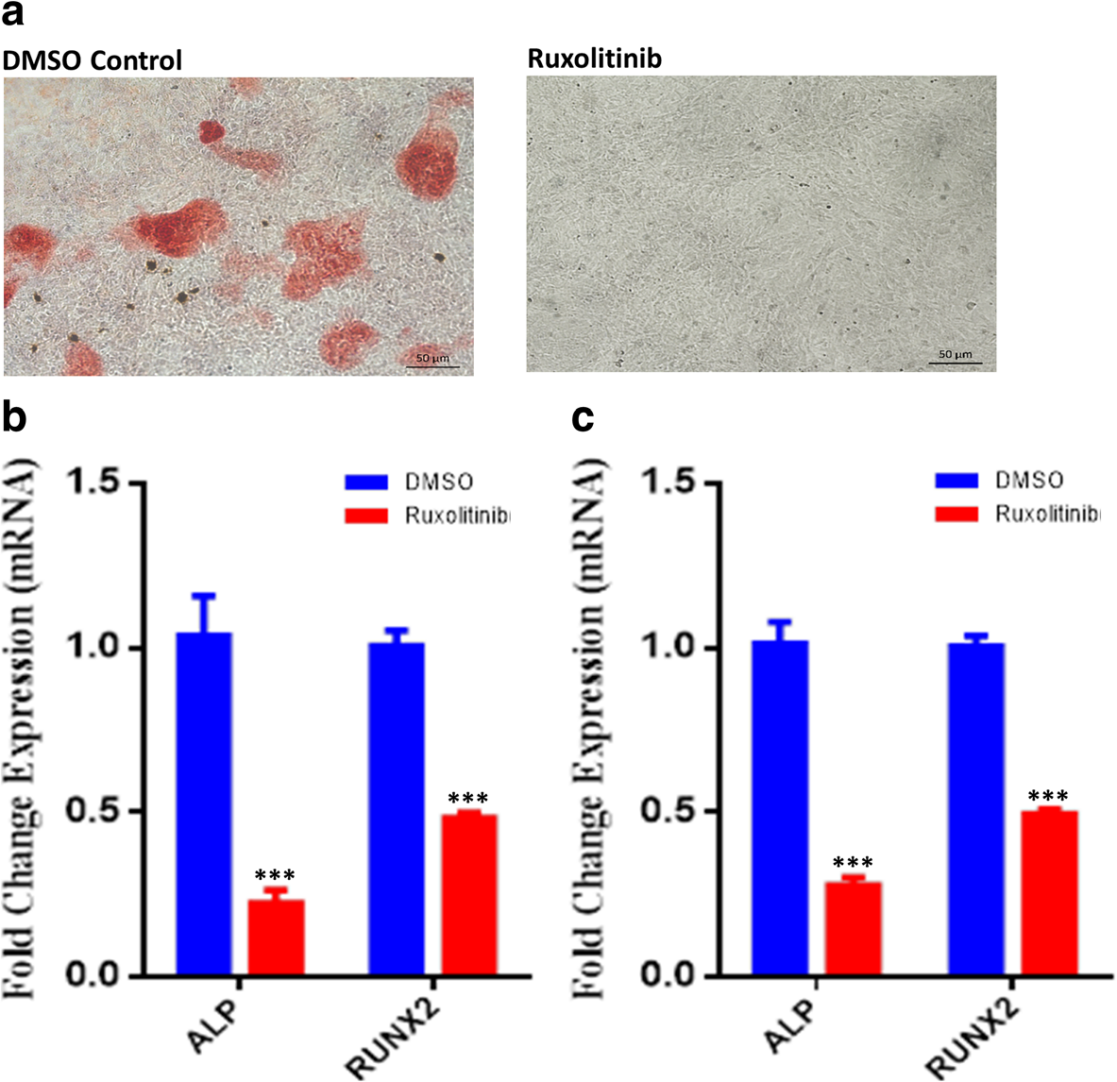
The effect of ruxolitinib on osteoblastic differentiation of hBMSCs. **a** hMSCs were induced into osteoblasts for 21 days in the absence (left panel) or presence (right panel) of ruxolitinib and were stained for mineralized matrix formation using Alizarin Red stain. Images were taken at × 10 magnification using a Zeiss inverted microscope. Quantitative RT-PCR analysis for gene expression of alkaline phosphatase (ALP) and RUNX2 in hBMSCs inducted into osteoblasts for 10 days (**b**) or 21 days (**c**) in the absence (blue) or presence (red) of ruxolitinib. Cells treated with DMSO were used as control. Gene expression was normalized to β-actin. Data are presented as mean fold change ± SEM (*n* = 6) from two independent experiments. ***P ≤ 0.0005. Abbreviations: ALP alkaline phosphatase, RUNX2 runt-related transcription factor 2, DMSO dimethyl sulfoxide

To assess the effects of ruxolitinib on mineralized matrix formation, hBMSCs were treated with ruxolitinib (3 μM) and induced into osteoblast for 21 days. Alizarin Red staining demonstrated significant reduction in mineralized matrix formation in ruxolitinib-treated hBMSCs compared to vehicle-treated controls (Fig. 3a). Similarly, ruxolitinib reduced the expression of ALP and RUNX2 osteoblast gene markers measured on day 10 (b) or day 21 (c) post-osteoblast induction.

### Ruxolitinib affects multiple signaling pathways during osteoblast differentiation of hBMSCs

**Fig. 4:**
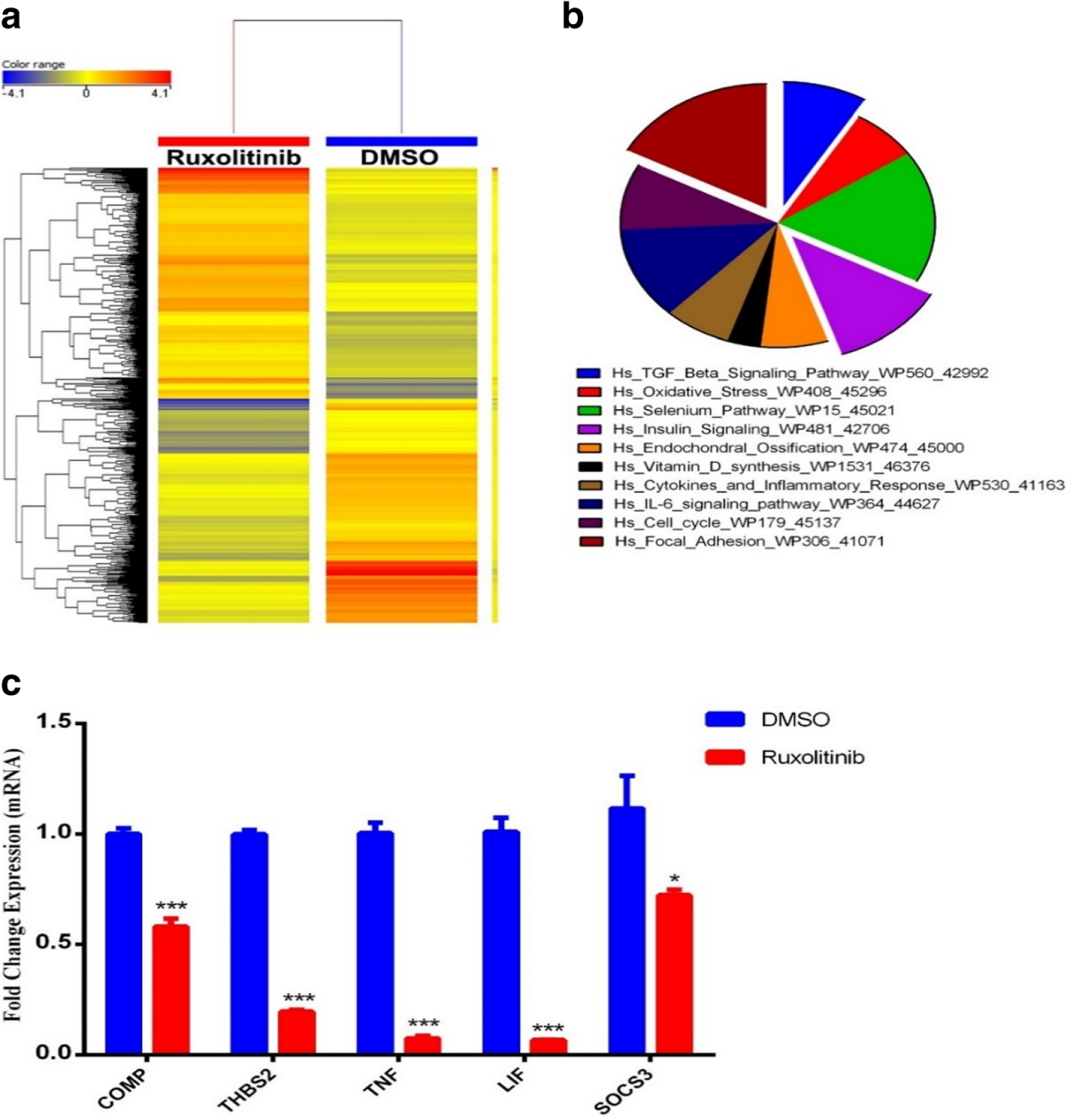
Ruxolitinib affects multiple pathways during osteoblastic differentiation of hBMSCs. **a** Heat map analysis and unsupervised hierarchical clustering performed on differentially expressed genes during osteoblast differentiation of ruxolitinib-treated compared to DMSO-treated control hBMSCs. **b** Pie chart illustrating the distribution of selected enriched pathway categories for the downregulated genes identified in osteoblast differentiated ruxolitinib-treated hBMSCs compared to DMSO-treated control cells. **c** Validation of a selected panel of downregulated genes during osteoblastic differentiation of ruxolitinib-treated hBMSCs compared to DMSO-treated control cells using qRT-PCR. Gene expression was normalized to β-actin. Data are presented as mean fold change ± SEM (*n* = 6) from two independent experiments; *P < 0.05; ***P < 0.0005

To understand the molecular mechanism by which ruxolitinib inhibits osteoblast differentiation of hBMSCs, we performed global gene expression profiling and pathway analysis comparing ruxolitinib-treated and DMSO-treated control cells, during osteoblast differentiation. Figure 4a shows the hierarchical clustering based on the differentially expressed genes and demonstrates clear separation of ruxolitinib-treated and DMSO (vehicle)-treated control cells. We identified 847 upregulated and 822 downregulated genes (fold change ≥ 2.0; *P* (corr) < 0.05) (Additional file 1). Pathway analysis of the downregulated genes revealed strong enrichment for several cellular processes involved in osteoblast differentiation (e.g., TGFβ signaling, insulin signaling, endochondral ossification, and focal adhesion). A number of significantly enriched pathways in ruxolitinib-treated cells are illustrated as a pie chart (Fig. 4b), wherein the size of the slice corresponds to fold enrichment. Among the identified pathways, TGFβ signaling, insulin signaling, and focal adhesion signaling were prominent. These genetic pathways are known for their role in regulating osteoblast differentiation of hBMSCs. A number of genes from the enriched pathways (TNF, LIF, SOCS3, COMP, and THBS2) were selected and validated using qRT-PCR, which collectively corroborated the microarray data (Fig. 4c).

### Effects of ruxolitinib on in vivo ectopic bone formation

**Fig. 5:**
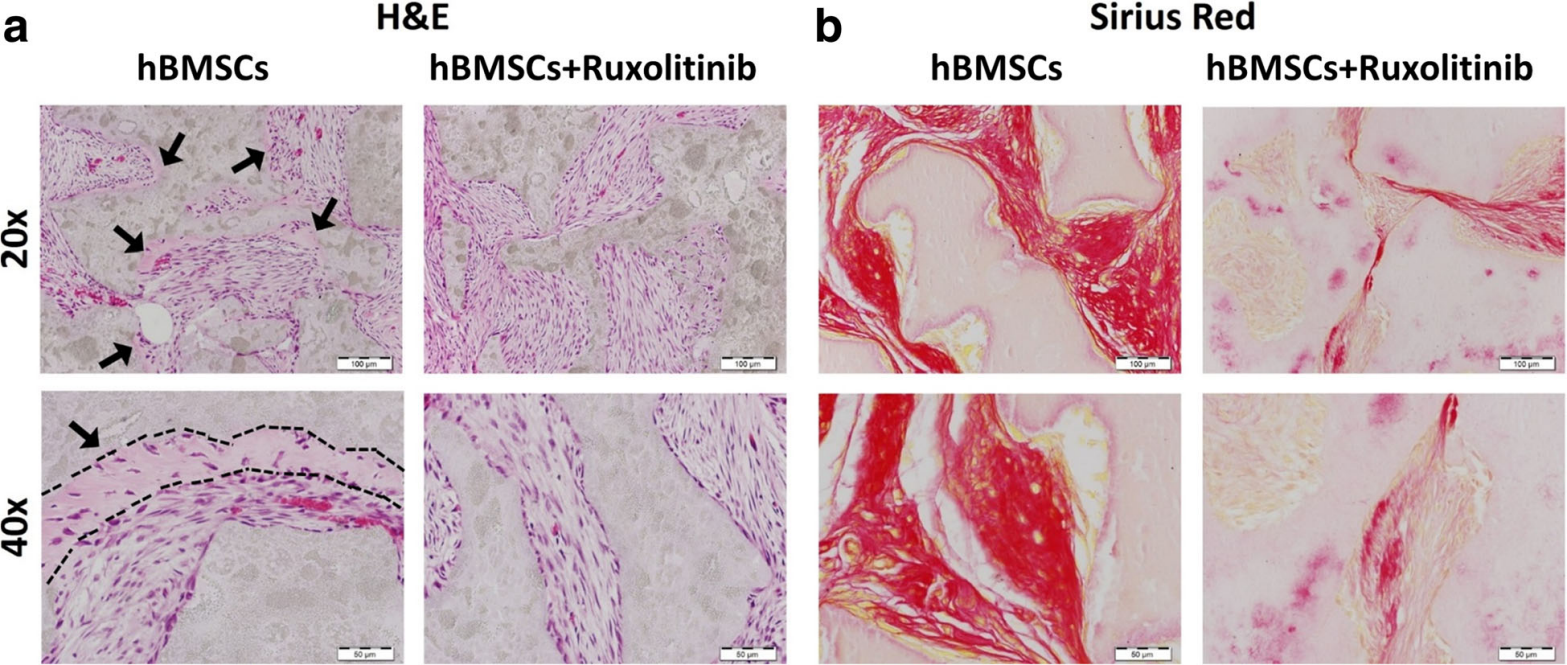
Ruxolitinib inhibits in vivo ectopic bone formation. Ruxolitinib-treated and control hBMSCs were implanted with hydroxyl apatite/tricalcium phosphate (HA/TCP) subcutaneously into NOD/SCID mice. The histology of in vivo bone formation was examined with H&E (**a**) and Sirius red (**b**) staining. Black arrows indicate the bone formation (× 20), and black line shows the bone formed zone with osteoblast between the HA and spindle-shaped hMSCs (× 40). Images were taken at × 20 (first row; scale bar = 100 μm) and × 40 (second row; scale bar = 50 μm) magnification using a light microscope. Abbreviation: H&E hematoxylin and eosin

To determine the regulatory role of ruxolitinib on in vivo bone formation, we implanted hBMSCs loaded on hydroxyapatite–tricalcium phosphate (HA/TCP) granules in the presence or absence of ruxolitinib into nude mice for 4 weeks. Histological analysis of the implants showed significant decrease in the formed ectopic bone in ruxolitinib-treated hBMSCs compared to control hBMSCs (Fig. 5a, b).

## Discussion

Small molecules, targeting specific signaling pathways, have recently emerged as a key tool to manipulate stem cell fate and differentiation potential in both mechanistic studies of stem cell biology as well as an approach to generate cells suitable for clinical use [6]. In the current study, we employed a well-characterized stem cell signaling library and performed unbiased functional screen on 73 small molecules targeting a number of signaling pathways relevant for hBMSC biology. These molecules covered a number of proteases, TGF-beta/Smad, JAK/STAT, Wnt, PI3K/Akt/mTOR, neuronal signaling, cell cycle, epigenetics, GPCR and G protein, Hedgehog, and GSK-3 inhibitors. Our initial screen identified several small molecule inhibitors, mainly targeting the JAK-STAT pathway, as potent inhibitors of osteoblastic differentiation of hBMSCs. In particular, ruxolitinib, a novel JAK-targeting small molecule inhibitor, was further studied and validated as a potent inhibitor of osteoblast differentiation.

JAK1 and JAK2 modulate the intracellular signaling of significant cytokines and growth factors for hematopoiesis and immune function including activation of signal transducers and activators of transcription (STAT). STAT3 is a ubiquitously expressed transcription factor activated by many cytokines and growth factors, including IL-6 family cytokines [16]. The receptors for the IL-6 family cytokines comprise of a ligand-binding subunit and a common signal-transducing subunit, gp130. Upon binding to their receptors, gp130 becomes activated leading to the activation of gp130-associated JAK (JAK1, JAK2, and TYK2) that subsequently lead to tyrosine phosphorylation of STAT3. Activated STAT3 localize to the nucleus and modulate various gene expression that regulate cell proliferation and differentiation in a cell-specific manner including bone metabolism [17]. The role of JAK-STAT signaling in osteoblastic differentiation is starting to unfold. For instance, inactivation and mutations of STAT3 in osteoblasts and osteocytes lead to distorted craniofacial and skeletal features, recurrent fractures, hyperextensible joints, reduce bone mass, strength, and load-driven bone formation, suggesting a role for STAT3 in osteoblast differentiation [18]. JAK-STAT signaling has been implicated in the maintenance of the stem cell pool in *Drosophila* and mammals [19]. Our findings from current study provide new evidence of potential involvement of JAK-STAT signaling in hBMSC biology.

Although in current study we did not investigate the downstream targets of ruxolitinib, it is well established that ruxolitinib is an ATP-competitive JAK1/2 inhibitor [20–22]. JAK1-deficient mice weighed less than their heterozygous and wild-type littermates, suggesting an important role for JAK1 in skeletal development [23]. Mouse embryonic fibroblasts derived from JAK2-deficient mice exhibited defects in signaling through a number of cytokine receptors, implying plausible role for JAK2 in skeletal development [24]. Cells treated with ruxolitinib exhibited diminished levels of phosphorylated STAT3, STA4, and STAT5 [25]. Therefore, it is plausible that ruxolitinib regulated osteoblastic differentiation of hBMSCs through inhibition of JAK-STAT3 signaling. Ruxolitinib might additionally inhibit other pathways known to be regulated by JAK, such as PI3K-AKT or ERK-JNK-p38, contributing to inhibition of osteoblastic differentiation [26, 27].

Global gene expression profiling of hMSC treated with ruxolitinib revealed multiple differentially regulated signaling pathways including TGFβ, insulin, endochondral ossification, and focal adhesion signaling, which are known to play an important role in regulating osteoblastic differentiation of hMSCs [28–33]. Those data are concordant with other published reports implicating protease-activated receptors [34], TGF-beta [2], Wnt/β-catenin [35], PI3K/Akt [36], cell cycle [37], and GPCR [38] signaling during osteogenesis.

Ruxolitinib is currently used in the clinic to treat patients with myelofibrosis, a clonal myeloproliferative neoplasm [39]. Ruxolitinib exhibited growth inhibition, apoptosis induction, and drop in inflammatory cytokine, mediated by inhibition of phosphorylate STAT via inhibition of JAK [20, 40]. No previous reports have been published regarding the effects of ruxolitinib on the osteoblast differentiation of hMSCs. We therefore investigated the expression of a selected panel of inflammatory cytokine (CXCL2, TNF, IL6, and CXCL1) from the microarray data during osteogenesis of hMSCs exposed to ruxolitinib and observed significant downregulation in the expression of those cytokines. While conceivable that small molecule inhibitors have specific targets, a number of studies have indicated deleterious effects of small molecule inhibitors on the biological function of mammalian cells [41, 42]. In particular, it was shown that hBMSCs are prone to cellular senescence under stress conditions [43]. Although we did not observe a significant change in cell viability of hBMSCs treated with ruxolitinib (3.0 μM) in the current study, it is plausible that inhibition of osteogenesis by ruxolitinib is in part due to possible effect on cellular senescence.

Enhancing bone formation and bone mass is needed in many conditions associated with bone loss such as in post-menopausal osteoporosis and glucocorticoid-induced osteoporosis, and suppression of osteogenic differentiation of hBMSCs by ruxolitinib may be relevant to a number of clinical conditions associated with ectopic bone formation or calcification including craniosynostosis and heart valve calcification [44, 45]. The clinical effectiveness of ruxolitinib in these conditions requires further studies.

## Conclusion

Our unbiased small molecule screen identified ruxolitinib, a JAK-STAT inhibitor, as potent inhibitor of osteoblastic differentiation of hBMSCs. Inhibition of bone formation by ruxolitinib might represent a novel therapeutic strategy for the treatment of pathological conditions caused by accelerated osteoblast differentiation and mineralization.

## Additional file

Additional file 1: List of differentially expressed genes (2.0 FC, p corr < 0.05) in human bone marrow mesenchymal stem cells (hBMSCs) differentiated into osteoblasts (day 10) in the presence of Ruxolitinib compared to DMSO. Differentially expressed genes (2.0 FC, p corr < 0.05) in human bone marrow mesenchymal stem cells (hBMSCs) differentiated into osteoblasts (day 10) in the presence of Ruxolitinib compared to DMSO detected using microarray. (XLSX 365 kb)

## Abbreviations

ALP: Alkaline phosphatase
ALZR: Alizarin Red
DMEM: Dulbecco’s modified Eagle’s medium
DMSO: Dimethyl sulfoxide
HA/TCP: Hydroxyapatite–tricalcium phosphate
hBMSCs: Human bone marrow stromal stem cell
hTERT: Human telomerase reverse transcriptase
JAK: Janus kinase
PBS: Phosphate-buffered saline
qRT-PCR: Quantitative reverse transcriptase-polymerase chain reaction
